# How Does Urban Farming Benefit Participants’ Health? A Case Study of Allotments and Experience Farms in Tokyo

**DOI:** 10.3390/ijerph18020542

**Published:** 2021-01-11

**Authors:** Kentaro Harada, Kimihiro Hino, Akiko Iida, Takahiro Yamazaki, Hiroyuki Usui, Yasushi Asami, Makoto Yokohari

**Affiliations:** 1 Urban & Spatial Development Headquarters, Nippon Koei Co., Ltd., Tokyo 102-8539, Japan; harada-kr@n-koei.jp; 2 Department of Urban Engineering, Graduate School of Engineering, The University of Tokyo, Tokyo 113-8656, Japan; iida@epd.t.u-tokyo.ac.jp (A.I.); yama3@g.ecc.u-tokyo.ac.jp (T.Y.); usui@ua.t.u-tokyo.ac.jp (H.U.); asami@csis.u-tokyo.ac.jp (Y.A.); myoko@edu.k.u-tokyo.ac.jp (M.Y.)

**Keywords:** urban farming, urban agriculture, allotment, experience farm, self-rated health, mental health, physical activity, city planning

## Abstract

In Japan, the world’s most rapidly aging country, urban farming is attracting attention as an infrastructure for health activities. In Tokyo, urban residents generally participate in two types of farming programs: allotments and experience farms. The availability of regular interaction among participants distinguishes these two programs. We quantitatively examined the difference in changes in self-reported health status between participants in these two types of urban farming. We obtained retrospective cross-sectional data from questionnaire surveys of 783 urban farming participants and 1254 nonparticipants and analyzed the data using ordinal logistic regressions. As a result, compared with nonparticipants, participants in both types of urban farming reported significantly improved self-rated health (SRH) and mental health (MH). After controlling for changes in their physical activity (PA), although participants in allotments did not report significant improvement in SRH and MH, those in experience farms did, suggesting that their health improvement was not only caused by an increase in PA but also by social interaction among participants. From the perspective of health promotion, public support is needed not only for the municipality’s allotments but also for the experience farms operated by the farmers themselves.

## 1. Introduction

### 1.1. The Role of City Planning in Improving Public Health

Modern city planning was initiated in the 19th century to improve public health through sanitation [1]. Meanwhile, the leading causes of death today in the developed world are lifestyle-related diseases [2]. Therefore, lifestyle changes are necessary to prevent such diseases and prolong healthy life expectancy [3]. This is a challenge that developed nations are commonly facing. In particular, Japan has the world’s most rapidly aging population (25.9% are aged 65 years or above) and needs to more effectively control its rising social security and national healthcare costs [4]. One of the government’s priorities is to reduce the need for long-term care by, e.g., encouraging older adults, who do not yet need long-term care, to exercise. In addition to improving physical health through exercise, the mental and social aspects of health should be considered [5].

It has been suggested that given these kinds of societal needs, city planning has an important role to play in the promotion of public health [6]. One initiative that has received considerable attention is providing city residents with opportunities to engage in urban gardening and farming [7,8,9]. The health effects (i.e., physical, mental, and social health) of these activities have been studied mainly in the Western society such as Europe and North America [10,11,12,13,14,15,16]; however, it is not yet clear whether similar effects exist in other diverse regions worldwide [7].

### 1.2. Urban Farming in Japan

Like many cities in the world, Japanese cities have expanded by encompassing the surrounding farming villages in the 20th century [17]. As a result, large amounts of agricultural land have significantly disappeared and have been converted into urban land uses. Meanwhile, city planning systems, including tax incentives on urban farmlands (e.g., the Productive Green Land Act), have created an environment in which farmers can continue to farm their land even after the area has been urbanized [18]. This has given Japanese cities a unique landscape that has a mixture of urban and agricultural land uses [19]. Even today, in the capital city of Tokyo, which has a population of 14 million people, approximately, 4000 hectares of farmland exist in urbanized areas (Figure S1).

Professional farmers mainly cultivate these urban farmlands, and farm products are sold through markets or directly to consumers at farm stands [20]. In addition, some urban farmlands are also used for recreational purposes by city residents. National government also emphasizes the promotion of urban agriculture as a means of supplying fresh farm products to city residents, but at the same time, urban farmland is considered important as a place to increase city residents’ understanding and interest in urban agriculture [21].

There are two major types of farming programs in which city residents can participate. The first is referred to in Japanese as shimin noen (literally, “citizens’ farm”). In this study, these are called “allotments” because this type of program is similar to allotment gardens in the United Kingdom. To create this type of farm, the municipal government, or other entity, rents the land from the farmer and then rents out lots to city residents. In Japan, because of the yearly growth in demand for allotments, a system for creating them has been implemented since 1989. In 2018, there were 434 allotments and 24,086 lots in Tokyo [22].

In the other type of program, referred to in Japanese as taiken noen (literally “experience farm”), the farmers themselves give city residents an opportunity to experience farming “hands-on,” using their land to teach participants how to farm. Similar to allotments, experience farms also allot parcels of land to the participants. However, legally speaking, the parcels are not rented but rather are simply being farmed by city residents who participate in a program directed by the farmer. The first farm of this type was opened in 1996 by a farmer in Tokyo’s Nerima Ward. In Tokyo, the number of experience farms has now grown to 111, with 6247 lots [22].

While allotments and experience farms both allot parcels to city residents for the purpose of farming, from the users’ perspective, they are very different. In allotment programs, the participants decide themselves how to cultivate their parcel of land, and everyone is free to farm as they wish. City residents are responsible for obtaining everything they need, including seeds, seedlings, and farming equipment. By contrast, on an experience farm, the farmer holds training sessions for participants several times a month to teach them the skills and knowledge they need to cultivate their parcel of land according to a cultivation plan determined by the farmer. Everything the participants need is provided by the farmer and shared among the participants. In addition, the farmer regularly hosts social events, such as a harvest festival. In other words, there is an important difference between the two programs in that, on allotments, participants work entirely independently, while on experience farms, participants work individually but are also provided with regular opportunities to socialize.

### 1.3. The Effects of Urban Farming on Health

Over the last few decades (before studies on urban gardening and farming began), a variety of epidemiological studies have been conducted regarding the effects of urban green spaces, i.e., parks, street trees, and gardens, on health. These studies have shown that the view of nature through windows [23,24,25], living in neighborhoods with a lot of greenery [26,27,28,29], and regularly experiencing nature [30,31,32,33,34] benefit people’s health and well-being in various ways.

Starting in the 1990s, qualitative and descriptive research began to suggest that urban allotment and community gardens, which are subtypes of urban green spaces, were also beneficial for people’s health and well-being [35,36,37,38,39,40,41,42]. Recently, the number of epidemiological studies on this topic has increased [43]. For example, it has been found that older adults that engage in urban gardening have better scores on health indicators, such as physical activity level [10,11] and self-rated health and life satisfaction [11], than those who do not engage in this activity. In addition, comparative studies of adult gardeners and nongardeners have indicated that gardening reduces the risk of being overweight or obese [12] and improves mental health factors such as self-esteem, mood [13], and life satisfaction [14], as well as physical and psychological well-being and social cohesion [7]. Other studies have found that compared with activities such as reading [15] or indoor exercise [16], gardening and farming are better for relieving stress.

All of these studies demonstrate that gardening and farming have many positive effects on the health and well-being of city residents. However, there are a wide variety of urban farming and gardening programs with various approaches. Therefore, various programs differ regarding the health benefits they may deliver, but studies have yet to focus on the differences among program types and assess what types of farming programs may deliver greater health benefits. In addition, most previous studies have focused only on health status at one point in time and did not investigate changes in health perceptions that are attributable to urban farming. It is important to focus on these changes to understand whether health benefits result from continuous farming activity.

### 1.4. Study Purpose

This study evaluates two typical types of farming programs available to Tokyo residents—allotments and experience farms—to determine (1) whether program participants have better perceptions of changes in health status compared with nonparticipants and (2) whether there are differences in health benefits according to program type. The study was conducted in Tokyo, where many farmers still have farmland within the dense urban fabric and where a variety of urban farming programs are taking place.

Because of the COVID-19 pandemic, even more attention is being paid to urban farming and/or gardening programs and their effects on the health of city residents who participate in them [44,45]. This study may provide important implications for postpandemic city planning and public health by identifying, which characteristics farming programs require to provide more benefits to participants’ health.

## 2. Methods

### 2.1. Target Farms

**Figure 1:**
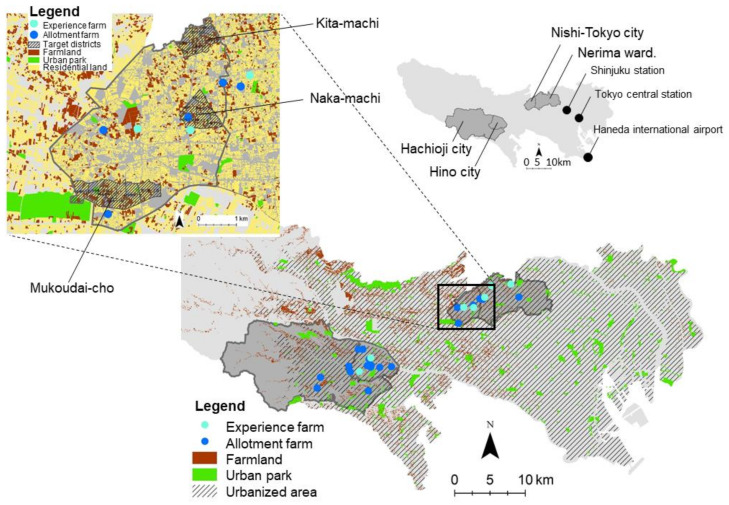
Locations of allotments and experience farms in Tokyo.

The survey was conducted in four municipalities between 10 and 50 km from central Tokyo, i.e., Nerima Ward and Nishitokyo City in the near suburb and Hino City and Hachioji City in the far suburb (Figure 1). We chose them after considering location, mix of agricultural and residential land uses, and the number of allotments and experience farms. The urban park area per person in these sites ranges from 1.3 to 11.7 m^2^ per person, smaller than in Western cities, but the area increases from 5.9 to 19.0 m^2^ per person when urban farmlands are included.

**Figure 2:**
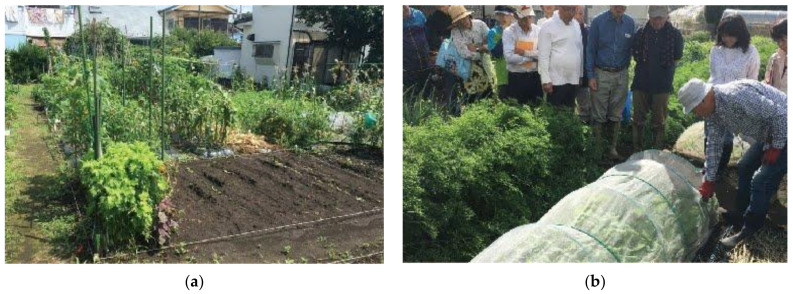
An allotment and an experience farm: (**a**) Midori-machi allotment, Hachioji City and (**b**) Tommy Club (experience farm), Nishi-Tokyo City. (Photographs: Kentaro Harada.).

A questionnaire survey was conducted at 26 farms in four municipalities: 19 allotments and 7 experience farms (Figure 2 and Table S1). We selected them to represent various lot sizes, numbers of lots, and land uses around them. The average annual expense for an experience farm is 42,000 yen (about 400 USD), compared with an allotment, which is about 7000 yen on average (about 65 USD). Each lot on experience farms is 30 m^2^, while allotments average 17 m^2^. Farm users of experience farms pay about six times more and cultivate about twice as much area as in allotments.

Along with the questionnaire survey, authors visited some allotments and all experience farms in four municipalities for observation surveys (e.g., an interview with participants while helping with cultivation) from late May to early November 2019.

### 2.2. Questionnaire Survey

We conducted questionnaire surveys to test the hypothesis that changes in health status differ between three groups—participants of allotments, experience farms, and the control group. The intervention group consisted of those aged 40 and above who participated in the target farms (Figure 1). The questionnaire for the intervention group was distributed to 1092 participants in 19 allotments and 626 participants in seven experience farms in October 2019. The responses from allotments participants were collected by mail and those from experience farm participants were collected directly.

The control group consisted of nonparticipants aged 40 and above, who lived in the three neighborhoods of Nishi-Tokyo City, where agricultural and residential land uses coexist (Figure 1). We chose these neighborhoods because we thought it appropriate to form a control group consisting of residents who live in neighborhoods with easy geographical access to urban agriculture but who do not participate in it. There are five allotments and five experience farms in the city [18], which are not sufficient for the population of 202,000. The questionnaires for the control group were distributed to 3000 randomly selected residents and were collected by mail in December 2019. Respondents who answered in the questionnaire that they had participated in urban farming were excluded from the analysis. In addition, we excluded respondents under the age of 40 and those who answered less than 80% of the required questions.

The study protocol was approved by the Ethical Committee of School of Engineering, The University of Tokyo (approval number KE19-41).

### 2.3. Variables and Statistical Analysis

We asked the respondents their self-rated health (SRH), mental health (MH), and physical activity (PA), which were commonly used in previous studies [46,47,48,49], with items such as “I am in good health,” “I have no anxiety, stress, or worries,” and “I have sufficient PA,” using a 4-point Likert scale ranging from “yes” to “no”. We also asked whether their SRH, MH, and PA had improved compared with how they were 3 years ago, using a 5-point Likert scale ranging from “better” to “worse.” The time period of 3 years was set, following previous studies [50,51]. The outcome variables were the changes in SRH, MH, and PA. Although we did not directly ask about social health among the three dimensions of health (i.e., physical, mental, and social health), it will be considered in discussions based on the analysis results.

The explanatory variables were participation in urban gardening (allotment, experience farm, and non-participation) and its interaction terms with gender and age (older, ≥70 or younger, and <70). We divided the participation group depending on whether they had participated for 3 years because changes in their SRH, MH, and PA were asked in comparison to 3 years ago. The control variables were gender, age, household (living alone or not), employment (employed or not), and the current status of each outcome variable (i.e., current SRH was entered as a control variable in the model for the change in SRH).

Ordinal logistic regression was used in each model for the three outcome variables—change in SRH, (model 1), change in MH (model 2), and change in PA (model 3). In addition, since PA affects SRH and MH [52,53], we also examined models 4 and 5 in which a change in PA was added to the control variables of models 1 and 2, respectively. Female, age less than 70 years, living alone, being unemployed, the last quartile of the present health status, and change in PA were set as the reference categories. The significance level was set at *p* < 0.05. All statistical analyses were conducted using IBM SPSS Statistics 25 (IBM Corp., Armonk, NY, USA).

## 3. Results

### 3.1. Descriptive Statistics

The respondents consisted of 540 allotment participants (response rate: 49.5%), 154 experience farm participants (24.6%), and 729 nonparticipants (24.3%). Table 1 presents the descriptive statistics of the questionnaire surveys. The sample comprised 56.0% male, 38.6% older adult, and 51.9% employed respondents, with 8.9% of the respondents living alone. Approximately, 60% of the participants in both experience farms and allotments had participated in such programs for more than 3 years. Compared with nonparticipants, urban farming participants were more likely to be male and over 70 years old. The percentages of respondents who answered they recently felt (rather) good in terms of SRH, MH, and PA were 82.7%, 64.1%, and 54.9%, respectively. The percentages of respondents who answered that they felt (rather) better recently than 3 years ago were 17.9% for SRH, 17.1% for MH, and 18.7% for PA.

### 3.2. Ordinal Logistic Regression

The results of the ordinal logistic regression analyses for changes in SRH, MH, and PA are presented in Table 2. The threshold indicates that the more positive the coefficient is, the better the health condition compared with the condition 3 years ago. In model 1, in relation to the outcome variable of change in SRH, the variables participation in urban farming, living alone, and current SRH were significant. With reference to nonparticipants, the change in SRH was significantly better in the order of experience farm participants, ≤3 years (B = 1.77; *p* < 0.001); experience farm participants, >3 years (B = 1.38; *p* < 0.001); allotment participants, >3 years (B = 0.74; *p* = 0.002); and allotment participants, ≤3 years (B = 0.65; *p* = 0.009). Although not significant at the 5% level, younger participants both in experience farms and allotments tended to improve their SRH. In model 2, in relation to the outcome variable of change in MH, the variables participation in urban farming, being older adults, and current MH were significant. With reference to nonparticipants, the change in MH was significantly better in the order of experience farm participants, ≤3 years (B = 1.35; *p* = 0.001); experience farm participants, >3 years (B = 0.73; *p* = 0.029); and allotment participants, >3 years (B = 0.67; *p* = 0.006), while not significant in allotment participants, ≤3 years. In model 3, in relation to the outcome variable of change in PA, the variables participation in urban farming, being older adults, being employed, and current PA were significant. With reference to nonparticipants, the change in PA was significantly better for experience farm participants, ≤3 years (B = 1.06; *p* = 0.006) and experience farm participants, >3 years (B = 0.79; *p* = 0.014), while not significant in allotment participants regardless of their years of participation.

The results of the ordinal logistic regression analyses for changes in SRH and MH with changes in PA added as a control variable are presented in Table 3. In model 4, where change in PA was added to the explanatory variables of model 1, participation in experience farms was significant (≤3 years: B = 1.52 and *p* < 0.001; >3 years: B = 1.39 and *p* < 0.001), whereas participation in allotments was not. The explanatory variables that were significant in models 1 and the change in PA were significant. Similarly, in model 5, where the change in PA was added to the explanatory variables of model 2, participation in the experience farm (≤3 years) was significant (B = 092; *p* = 0.024), whereas participation in experience farm (>3 years) and allotments were not. The explanatory variables that were significant in models 2, as well as the change in PA, were significant.

## 4. Discussion

The results confirmed that participants in urban agriculture improved in terms of SRH, MH, and PA compared with nonparticipants, which support the findings from previous studies [7,10,11,12,13,14]. Furthermore, we found a difference in the degree of improvement depending on the type of urban farming. First, the SRH of experience farm participants (especially those with less than 3 years of participation) significantly improved compared with that of allotment participants. This may be because even beginners can grow vegetables well and enjoy farming with the help of experienced farmers. Second, the MH of the experience farm participants also improved significantly more than that of allotment participants, and allotment participants who had participated for less than 3 years did not show a significant difference from the nonparticipants. In contrast to experience farms, allotment participants may not be able to cultivate well when they first join the program, and it may take them some time to improve. Third, the PA of experience farm participants was significantly improved compared with that of nonparticipants, while the PA of allotment participants was not significantly improved. This may be because the experience farm participants cultivated about twice as much plot area as that cultivated by allotment participants. Experience farm participants also cultivated more intensively and were more productive per unit area [54].

Moreover, the SRH of experience farm participants significantly improved compared with that of nonparticipants, even when controlling for changes in PA, while the SRH of allotment participants did not significantly improve. This suggests that participation in experience farms has health benefits other than an increase in PA. In a qualitative study based on interviews with older adults participating in agricultural activities, positive effects, such as “joy of interaction with other participants” and “going out with other participants,” were found on the mental and social health of the participants who interacted with other participants [41]. Our results suggest a positive health impact of such interactions (which characterize experience farms) among participants. The improvement in MH for experience farm participants (having less than 3 years of participation) may be for the same reason.

### 4.1. Suggestion from a Public Health Perspective

Participants aged above 70 years had worse PA and MH score than younger participants. Although deterioration in health status with age is a natural process, participation in urban farming might extend healthy life expectancy [13]. In addition, people who live alone had worse SRH compared with people who live with others. It is known that lifelong unmarried people have a shorter life expectancy than those who are married [55]. Urban farming activities, especially the interactions among participants, which typically occur on experience farms, could mitigate the deteriorating health status of single people. To encourage older adults and single people to be more actively involved in urban farming, not only conventional allotments and experience farms, but also community garden-type farms that are typically seen in Western societies [12,35,36,37,38,39] might be effective, where participants cultivate farms together instead of cultivating lots individually. In such farms, the organizer is required to have a high level of management skills, but it is also possible to share the workload according to the physical ability of the participants and to distribute the harvest according to family size; therefore, older adults and single people can participate more easily.

### 4.2. Suggestion from a City Planning Perspective

The management of urban farmland is a method of solving diverse urban problems [56] such as crime [57] and food justice issues [58]. It is not easy to conserve and manage urban farmland in a dense urban area, but it was shown that urban residents’ use of farms can improve public health, which was the original goal of modern city planning. Furthermore, for postpandemic city planning, the role of farms in solving urban problems has become even more important [45,46]. This study showed that the health effects of the farms differed by type of farm, with experience farms having a higher effect than allotments. However, the establishment and management of experience farms usually depend on the intentions of landowners. The future society with a super-aging population needs health-centered city planning. This can be achieved by encouraging local governments to convert into experience farms the remaining land in areas with a large number of older adults and single households and by providing public support for the establishment and operation of experience farms. Some advanced municipalities, such as Nerima Ward and Hino City in Tokyo (Table S2), already provide subsidies to users or owners of experience farms, and such efforts may be effective in expanding and developing them.

### 4.3. Limitation of the Study

This study makes an important contribution to existing research on the health benefits of urban farming, but it has some limitations. First, although urban farms were categorized into two types depending on whether opportunities for interaction between participants exist, there are differences in farm size, equipment, and service level, even within the same type. Future studies should take such differences into account based on detailed surveys.

In addition, our survey asked participants about their subjective health status in a cross-sectional survey, where they answered retrospectively about changes in their health status. In this regard, health benefits based on objective and longitudinal measurements of health should be examined [59].

## 5. Conclusions

This study examined the difference in health changes between participants in allotments and experience farms. Participants in both types of urban farming reported significantly improved SRH and MH compared with those reported by nonparticipants. Even after controlling for changes in their PA, participants in experience farms reported significant improvement in SRH and MH, while participants in allotments did not. The results imply that their health improvement may not only be attributed to an increase in PA but also to the social interactions among participants and with the farmers. This result indicates that from a health promotion perspective, public support is needed not only for the municipality’s allotment programs, but also for the experience farm programs operated by farmers. Moreover, the variables being older and living alone were negatively associated with health indices. To encourage these people to participate in farming activities and prolong their healthy life expectancy, community garden-type farming, where participants can work together, would be effective, especially in aging countries like Japan.

## Figures and Tables

**Table 1:** Descriptive statistics of respondents (*n* = 1254).

Variables	Options	Experience Farm		Allotment		Non-Participant		Total	
		*n*	%	*n*	%	*N*	%	*N*	%
Gender	Male	78	59.1	345	73.2	279	42.9	702	56.0
	Female	54	40.9	126	26.8	372	57.1	552	44.0
Age	40s	11	8.3	39	8.3	131	20.1	181	14.4
	50s	25	18.9	44	9.3	162	24.9	231	18.4
	60s	48	36.4	152	32.3	158	24.3	358	28.5
	70s	46	34.8	197	41.8	146	22.4	389	31.0
	80 or older	2	1.5	39	8.3	54	8.3	95	7.6
Living alone	Yes	9	6.8	34	7.2	68	10.4	111	8.9
	No	123	93.2	437	92.8	583	89.6	1143	91.1
Employment	Employed	64	48.5	205	43.5	382	58.7	651	51.9
	Unemployed	68	51.5	266	56.5	269	41.3	603	48.1
Years of participation	>3 years	83	62.9	286	60.7			471	61.2
	Others	49	37.1	185	39.3			132	38.8
Good SRH	No	6	4.5	31	6.6	40	6.1	77	6.1
	Rather no	11	8.3	48	10.2	81	12.4	140	11.2
	Rather yes	63	47.7	232	49.3	340	52.2	635	50.6
	Yes	52	39.4	160	34.0	190	29.2	402	32.1
Change in SRH	Worse	3	2.3	18	3.8	39	6.0	60	4.8
	Rather worse	9	6.8	84	17.8	146	22.4	239	19.1
	Not changed	81	61.4	278	59.0	371	57.0	730	58.2
	Rather better	19	14.4	53	11.3	65	10.0	137	10.9
	Better	20	15.2	38	8.1	30	4.6	88	7.0
Good MH	No	18	13.6	66	14.0	141	21.7	225	17.9
	Rather no	38	28.8	130	27.6	172	26.4	340	27.1
	Rather yes	58	43.9	206	43.7	267	41.0	531	42.3
	Yes	18	13.6	69	14.6	71	10.9	158	12.6
Change in MH	Worse	4	3.0	21	4.5	49	7.5	74	5.9
	Rather worse	15	11.4	49	10.4	112	17.2	176	14.0
	Not changed	85	64.4	310	65.8	395	60.7	790	63.0
	Rather better	17	12.9	58	12.3	71	10.9	146	11.6
	Better	11	8.3	33	7.0	24	3.7	68	5.4
Sufficient PA	No	15	11.4	43	9.1	94	14.4	152	12.1
	Rather no	31	23.5	89	18.9	178	27.3	298	23.8
	Rather yes	57	43.2	199	42.3	233	35.8	489	39.0
	Yes	29	22.0	140	29.7	146	22.4	315	25.1
Change in PA	Worse	2	1.5	14	3.0	35	5.4	51	4.1
	Rather worse	15	11.4	81	17.2	128	19.7	224	17.9
	Not changed	83	62.9	281	59.7	380	58.4	744	59.3
	Rather better	19	14.4	49	10.4	71	10.9	139	11.1
	Better	13	9.8	46	9.8	37	5.7	96	7.7
Total		132	100.0	471	100.0	651	100.0	1254	100.0

Note: SRH means self-rated health; PA, physical activity; and MH, mental health.

**Table 2:** The results of the ordinal logistic regression analyses (models 1–3).

Variables	Model 1 Change in SRH		Model 2 Change in MH		Model 3 Change in PA	
	B (95% CI)	*p*	B (95% CI)	*p*	B (95% CI)	*p*
Threshold						
Worse/Rather worse	−4.38 (−4.84, −3.93)	**<0.001**	−4.9 (−5.44, −4.36)	**<0.001**	−4.89 (−5.38, −4.41)	**<0.001**
Rather worse/not changed	−2.15 (−2.51, −1.78)	**<0.001**	−3.22 (−3.71, −2.73)	**<0.001**	−2.73 (−3.13, −2.33)	**<0.001**
Not changed/rather better	0.99 (0.65, 1.33)	**<0.001**	0.56 (0.13, 0.98)	**0.011**	0.56 (0.2, 0.92)	**0.002**
Rather better/better	2.11 (1.74, 2.49)	**<0.001**	1.93 (1.47, 2.4)	**<0.001**	1.68 (1.29, 2.06)	**<0.001**
Participation in urban farming (Ref: nonparticipant)						
Experience farm, ≤3 years	1.77 (1.03, 2.51)	**<0.001**	1.35 (0.58, 2.12)	**0.001**	1.06 (0.31, 1.81)	**0.006**
Experience farm, >3 years	1.38 (0.76, 2)	**<0.001**	0.73 (0.08, 1.38)	**0.029**	0.79 (0.16, 1.42)	**0.014**
Allotment, ≤3 years	0.65 (0.17, 1.14)	**0.009**	0.35 (−0.15, 0.84)	0.175	0.18 (−0.31, 0.67)	0.468
Allotment, >3 years	0.74 (0.28, 1.21)	**0.002**	0.67 (0.19, 1.15)	**0.006**	0.44 (−0.03, 0.9)	0.064
Gender (Ref: female)		.		.		.
Male	−0.18 (−0.5, 0.15)	0.284	−0.21 (−0.54, 0.12)	0.215	−0.16 (−0.48, 0.16)	0.334
Age (Ref: younger)		.		.		.
Older (≥70)	−0.13 (−0.5, 0.24)	0.493	−0.45 (−0.84, −0.07)	**0.021**	−0.46 (−0.83, −0.08)	**0.017**
Living alone (Ref: no)		.		.		.
Yes	−0.49 (−0.88, −0.1)	**0.014**	0.24 (−0.16, 0.64)	0.239	−0.2 (−0.6, 0.2)	0.322
Employment (Ref: unemployed)		.		.		.
Employed	−0.06 (−0.32, 0.2)	0.643	−0.07 (−0.33, 0.19)	0.605	0.3 (0.04, 0.56)	**0.023**
Good SRH (Ref: yes)		.		.		.
No	−3.5 (−4.01, −2.98)	**<0.001**				
Rather no	−2.22 (−2.62, −1.82)	**<0.001**				
Rather yes	−0.8 (−1.06, −0.54)	**<0.001**				
Good MH (Ref: yes)						
No			−3.49 (−3.96, −3.02)	**<0.001**		
Rather no			−2.15 (−2.58, −1.72)	**<0.001**		
Rather yes			−0.49 (−0.86, −0.13)	**0.008**		
Sufficient PA (Ref: yes)						
No					−3.25 (−3.67, −2.82)	**<0.001**
Rather no					−2.13 (−2.48, −1.77)	**<0.001**
Rather yes					−0.87 (−1.17, −0.58)	**<0.001**
Interaction terms						
Experience farm × male	−0.4 (−1.17, 0.37)	0.306	−0.47 (−1.27, 0.33)	0.252	−0.34 (−1.12, 0.45)	0.399
Allotment × male	−0.07 (−0.59, 0.45)	0.791	−0.17 (−0.7, 0.36)	0.536	−0.22 (−0.73, 0.3)	0.417
Experience farm × older	−0.72 (−1.53, 0.08)	0.078	−0.28 (−1.11, 0.56)	0.514	0.01 (−0.8, 0.81)	0.99
Allotment × older	−0.46 (−0.96, 0.04)	0.069	0.18 (−0.33, 0.7)	0.481	0.12 (−0.38, 0.62)	0.64

Note: SRH means self-rated health; MH, mental health; PA, physical activity; and Bold, significant at the 5% level.

**Table 3:** The results of the ordinal logistic regression analyses (models 4 and 5).

Variables	Model 4		Model 5	
	Change in SRH		Change in MH	
	B (95% CI)	*p*	B (95% CI)	*p*
Threshold				
Worse/rather worse	−8.49 (−9.19, −7.78)	**<0.001**	−7.81 (−8.54, −7.08)	**<0.001**
Rather worse/not changed	−5.85 (−6.46, −5.24)	**<0.001**	−5.96 (−6.64, −5.28)	**<0.001**
Not changed/rather better	−1.92 (−2.45, −1.38)	**<0.001**	−1.65 (−2.23, −1.07)	**<0.001**
Rather better/better	−0.28 (−0.79, 0.22)	0.275	0.1 (−0.47, 0.67)	0.741
Participation in urban farming (Ref: nonparticipant)				
Experience farm, ≤3 years	1.52 (0.72, 2.33)	**<0.001**	0.92 (0.12, 1.71)	**0.024**
Experience farm, >3 years	1.39 (0.72, 2.06)	**<0.001**	0.55 (−0.12, 1.21)	0.107
Allotment, ≤3 years	0.39 (−0.13, 0.9)	0.141	0.16 (−0.36, 0.68)	0.546
Allotment, >3 years	0.46 (−0.03, 0.96)	0.065	0.39 (−0.1, 0.89)	0.121
Gender (Ref: female)		.		.
Male	−0.1 (−0.44, 0.24)	0.553	−0.15 (−0.49, 0.2)	0.4
Age (Ref: younger)		.		.
Older (≥70)	−0.04 (−0.44, 0.35)	0.828	−0.44 (−0.84, −0.04)	**0.03**
Living alone (Ref: no)		.		.
Yes	−0.53 (−0.94, −0.12)	**0.011**	0.19 (−0.23, 0.62)	0.365
Employment (Ref: unemployed)		.		.
Employed	−0.16 (−0.43, 0.11)	0.249	−0.22 (−0.49, 0.06)	0.12
Good SRH (Ref: yes)		.		.
No	−3.04 (−3.59, −2.49)	**<0.001**		
Rather no	−1.86 (−2.28, −1.43)	**<0.001**		
Rather yes	−0.64 (−0.92, −0.36)	**<0.001**		
Good MH (Ref: yes)				
No			−3.37 (−3.86, −2.88)	**<0.001**
Rather no			−2.09 (−2.54, −1.64)	**<0.001**
Rather yes			−0.41 (−0.8, −0.01)	0.043
Change in PA (Ref: better)				.
Worse	−6.34 (−7.12, −5.56)	**<0.001**	−4.44 (−5.17, −3.71)	**<0.001**
Rather worse	−4.9 (−5.47, −4.34)	**<0.001**	−3.43 (−3.97, −2.89)	**<0.001**
Not changed	−3.53 (−4.01, −3.04)	**<0.001**	−2.65 (−3.11, −2.18)	**<0.001**
Rather better	−1.6 (−2.14, −1.07)	**<0.001**	−0.81 (−1.35, −0.28)	**0.003**
Interaction terms		.		.
Experience farm × male	−0.49 (−1.33, 0.34)	0.248	−0.33 (−1.17, 0.5)	0.434
Allotment × male	0.08 (−0.47, 0.63)	0.776	−0.03 (−0.59, 0.52)	0.913
Experience farm × older	−0.81 (−1.67, 0.06)	0.068	−0.38 (−1.25, 0.49)	0.396
Allotment × older	−0.5 (−1.03, 0.02)	0.061	0.23 (−0.3, 0.77)	0.395

Note: SRH means self-rated health; MH, mental health; PA, physical activity; and bold, significant at the 5% level.

## Data Availability

The data are not publicly available due to a confidentiality agreement with survey respondents.

## Supplementary Materials

The following are available online at https://www.mdpi.com/1660-4601/18/2/542/s1, Figure S1: Urbanized area and farmland in Tokyo, Table S1: List of target farms, and Table S2: Measures by which municipalities support experience farms.
