# Supplementary material for: How Does Urban Farming Benefit Participants’ Health? A Case Study of Allotments and Experience Farms in Tokyo
Source: Int J Environ Res Public Health. 2021 Jan 11;18(2):542. doi: 10.3390/ijerph18020542 (PMC7826565; doi:10.3390/ijerph18020542)
Supplement: Supplementary file 1 [file ijerph-18-00542-s001.pdf]

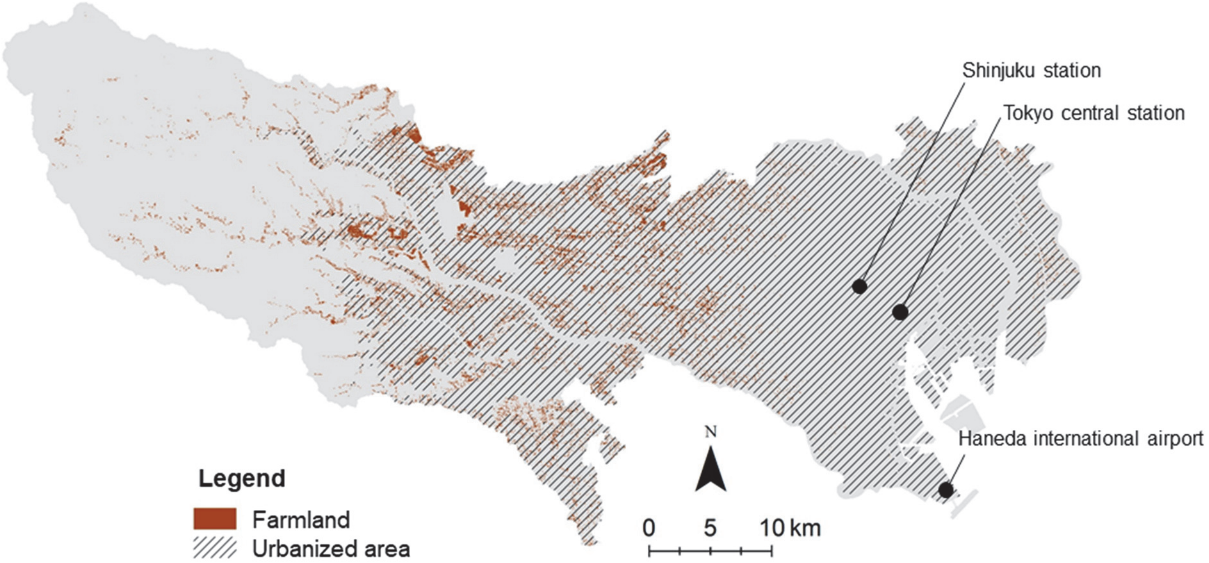

Figure S1: Urbanized area and farmland in Tokyo

Table S1. List of target farms

Type	No.	Name	Municipality	Annual expense (yen/lot*)	Lot size (m^2/lot)	Number of lots	Sample size
Allotments	1	Minamioizumisan-chome Kumin Noen	Nerima Ward	4,800	15	42	12
	2	Takamatsusan-chome Kumin Noen				38	20
	3	Minamioizumi Shimin Noen		19,200	30	48	26
	4	Shin-machi Shimin Noen	Nishitokyo City	5,000	12	93	41
	5	Nishihara Shimin Noen				53	18
	6	Naka-machi Shimin Noen				54	23
	7	Shin-Atagoshita Nishi Shimin Noen	Hino City	6,000	20	62	30
	8	Shin-Sakashita Shimin Noen				85	33
	9	Bontenyama Nishi Shimin Noen				50	24
	10	Ochikawa Shimin Noen				63	15
	11	Minamidaira Shimin Noen				20	6
	12	Higashitoyoda Shimin Noen				48	20
	13	Mukaigawara Shimin Noen				82	34
	14	Nanatsuzuka Nishi Shimin Noen				60	23
	15	Asahigaoka Shimin Noen				82	38
	16	Misawa Shimin Noen				67	25
	17	Midori-machi Noen	Hachioji City	7,500	10	45	22
	18	Terada Noen				36	23
	19	Koshino Noen				64	14
Experience farms	20	Iga-san no Hatake	Nerima Ward	50,000	30	122	28
	21	Midori no Sanpomichi				135	21
	22	Minamioizumi Yasaimura				140	17
	23	Kitappara	Nishitokyo City	46,000	30	95	21
	24	Tommy Club		54,000	30	116	33
	25	Kobasan Noen	Hino City	40,000	30	36	22
	26	Kishino Noen				22	12

* One yen is about 0.96 U.S. dollars in December 2020.

Table S2. Measures by which municipalities support experience farms

Municipality	Support for experience farms by municipalities
Nerima Ward	The municipality subsidizes 12,000 yen* per lot for participants living in Nerima Ward.
Nishitokyo City	No official support is provided.
Hino City	For the first three years after the opening of an experience farm, the municipality provides 10,000 yen* per lot to farm owners.
Hachioji City	No official support is provided.

* One yen is about 0.96 U.S. dollars in December 2020.
